# Case Report: Imerslund Grasbeck syndrome: a rare cause of megaloblastic anemia in a well-nourished child

**DOI:** 10.3389/fnut.2026.1883519

**Published:** 2026-08-13

**Authors:** Ritika Khurana, Purva Kanvinde, Sangeeta Mudaliar

**Affiliations:** Department of Paediatric Hematology-Oncology, Bai Jerbai Wadia Hospital for Children, Mumbai, India

**Keywords:** *AMN* gene, *CUBN* gene, Imerslund Grasbeck, inherited vitamin B12 deficiency, megaloblastic anemia

## Abstract

Megaloblastic anemia, characterized by macrocytic anemia, is most commonly caused by nutritional vitamin B12 deficiency; however, inherited disorders of cobalamin absorption should be considered in children with adequate dietary intake. One such disorder is Imerslund–Gräsbeck syndrome (IGS), a rare autosomal recessive condition characterized by selective intestinal malabsorption of vitamin B12 due to mutations in the *CUBN* or *AMN* genes. We report a 7-year-old boy, born of a third-degree consanguineous marriage, who presented with generalized rash, easy fatigability, and recurrent oral ulceration. Examination revealed pallor and hyperpigmented knuckles without organomegaly. Growth parameters were between the 10th and 25th centiles. Investigations showed severe macrocytic anemia (hemoglobin 2.5 g/dL), leukopenia, thrombocytopenia, markedly reduced vitamin B12 levels (<50 pg./mL), and peripheral smear findings of macrocytosis with hypersegmented neutrophils. In view of adequate nutritional intake, further evaluation for malabsorption was undertaken. There was no clinical evidence of gastrointestinal malabsorption, and anti-intrinsic factor antibody testing was negative. Urinalysis demonstrated mild proteinuria. Genetic analysis identified a homozygous mutation in the *AMN* gene, confirming the diagnosis of IGS. The child started on parenteral vitamin B12 therapy with plans for lifelong supplementation at intervals guided by clinical response. IGS should be suspected in children with megaloblastic anemia despite adequate dietary intake, particularly in the presence of proteinuria, parental consanguinity, or a poor response to oral supplementation. Early diagnosis and timely initiation of lifelong vitamin B12 therapy are essential to ensure optimal hematological recovery, growth, and neurodevelopmental outcomes.

## Highlights

- Look beyond nutritional causes in patients with a dietary history suggestive of adequate intake, parental consanguinity, or poor response to routine therapy.
- Sequential evaluation to rule out different causes of inherited vitamin B12 deficiency should be considered.

## 1 Introduction

Megaloblastic anemia is characterized by the presence of macrocytes in the peripheral blood and megaloblasts in the bone marrow. Vitamin B12 deficiency due to inadequate nutritional intake is the most common cause, followed by malabsorption disorders. One such disorder is Imerslund–Gräsbeck syndrome (IGS), a rare condition characterized by selective malabsorption of cobalamin. It may present with megaloblastic anemia, intermittent proteinuria, and failure to thrive.

### 1.1 Case description

A 7-year-old boy, born of a third-degree consanguineous marriage, presented with a history of a generalized rash, easy fatigability, and recurrent oral ulceration, noticed for the past 20 days. He was consuming an adequate diet for his age, including non-vegetarian food. There was no history suggestive of malabsorption, developmental delay, or cognitive deficits. There was no past or family history of blood transfusions. On examination, the child was pale and had hyperpigmented knuckles, with no organomegaly. His weight and height were between the 10th and 25th centiles, well below the projected mid-parental height. Neurological examination was normal.

### 1.2 Diagnostic evaluation

Baseline investigations (Table 1) were suggestive of macrocytic anemia with pancytopenia and vitamin B12 < 50 pg./mL. In view of an adequate dietary history, further evaluation was undertaken. Anti-intrinsic factor antibody testing was negative. Urinalysis revealed mild proteinuria. Exome-sequencing revealed a biallelic nonsense mutation c.862C > T (p. Gln288Ter) in exon 9 of *AMN* (NM_030943.4), confirming the molecular diagnosis of Imerslund–Gräsbeck syndrome. This variant is not found in any population databases and is classified as likely pathogenic (PVS1_VS and PM2_S) as per ACMG/AMP 2015 guidelines. The variant has been submitted to ClinVar as pathogenic (RCV003863217.4), and it has not been reported previously.

**Table 1:** Baseline and follow-up investigations.

Parameters	At diagnosis	2 weeks	1 year of oral therapy
Hemoglobin, MCV	2.5 g/dL, 101	9.7 g/dL, 95	13.8 g/dL, 79
White blood count	2,600 cells/microL	3,600 cells/microL	15,950
ANC/ALC^*^	494/2,054 cells/microL	1,456/3,024 cells/microL	11,310/3,960 cells/microL
Platelet count	65,000 per microL	3,64,000 per microL	2,59,000 per microL
Reticulocyte count	1.53%	2.2%	
LDH	846 U/L	210	
Vitamin B12 level	<50 pg./mL	–	640
Peripheral smear	Macrocytosis, hypersegmented neutrophils	–	

*ANC, Absolute neutrophil count; ALC, Absolute lymphocyte count.

### 1.3 Treatment and follow-up

The child was started on parenteral vitamin B12 and was planned to continue on lifelong vitamin B12 therapy. Parenteral vitamin B12 was administered monthly for 6 months, during which the child maintained hemoglobin and had no other symptoms of vitamin B12 deficiency. Following this, the child was started on high-dose oral vitamin B12 (methylcobalamin) 750 μg/day, on which the child has maintained parameters well over the subsequent 2 years. He is on regular follow-up, growing well, and there is no proteinuria at present.

## 2 Discussion

Megaloblastic anemia despite an adequate vitamin B12-containing diet, anemia unresponsive to oral supplementation, parental consanguinity, and symptoms suggestive of malabsorption should prompt evaluation beyond nutritional causes.

Imerslund–Grasbeck syndrome, first described independently by Imerslund and Grasbeck in 1960 (1, 2), is an autosomal recessive disorder characterized by vitamin B12 malabsorption due to mutations in the *CUBN* or *AMN* gene. IGS is a rare disorder with an estimated prevalence of less than 6 per 1,000,000 (3). The Vitamin B12-IF receptor consists of two subunits: cubilin and amnionless, together known as the cubam complex. This complex is essential for the intestinal absorption of vitamin B12 and for renal protein absorption. Retention of cubilin and amnionless within the endoplasmic reticulum of proximal tubular cells results in the absence of cubam on the cell surface. Consequently, albumin reabsorption in the proximal tubule is impaired, leading to proteinuria (3).

In a review by Kingma et al. involving 456 patients across the world with IGS, the median age at symptom onset was 2.3 years (range: 0–38 years), while the median age at diagnosis was 7.2 years, similar to our patient, who had early features of failure to thrive and short stature but was diagnosed only at 7 years of age (4). Non-hematological manifestations included gastrointestinal symptoms in 32% of patients, neurological manifestations such as fatigue and sensory or motor deficits in 16%, and respiratory symptoms in 3% (4). Our patient did not have convincing neurological or gastrointestinal symptoms, which are common in these patients and lead to evaluation beyond nutritional vitamin B12 deficiency. Unlike most studies in the literature, proteinuria in our patient had resolved on vitamin B12 treatment (5). The genetic variant in our patient is novel, but considering the strength of the type of mutation as well as subtle pointers in the child’s evaluation, the diagnosis was established and therapy initiated.

## 3 Conclusion

IGS should be considered in patients with macrocytic anemia, vitamin B12 deficiency, and persistent proteinuria (6). Most patients respond well to lifelong monthly parenteral vitamin B12 therapy. Some patients may also respond to high-dose oral vitamin B12 supplementation, as a small portion of vitamin B12 is absorbed passively. Early diagnosis and timely intervention are essential to support normal growth and neurological development.

## Data Availability

The datasets presented in this article are not readily available because of ethical and privacy restrictions. Requests to access the datasets should be directed to the corresponding author.
